# Magnetic properties of a non-centrosymmetric polymorph of FeCl_3_†

**DOI:** 10.1039/d4ma00635f

**Published:** 2025-06-02

**Authors:** Joshua J. B. Levinsky, Ankit Labh, Vladimir Pomjakushin, Uwe Keiderling, Alexander C. Komarek, Li Zhao, Jacob Baas, Catherine Pappas, Graeme R. Blake

**Affiliations:** a Zernike Institute for Advanced Materials, University of Groningen Nijenborgh 3 9747AG Groningen The Netherlands g.r.blake@rug.nl; b EaStCHEM School of Chemistry and Centre for Science at Extreme Conditions, University of Edinburgh Joseph Black building, David Brewster Road EH9 3FJ Edinburgh UK; c Faculty of Applied Sciences, Delft University of Technology Mekelweg 15 2629JB Delft The Netherlands; d Department of Condensed Matter Physics, Charles University Ke Karlovu 5 121 16 Praha 2 Czechia; e TUM School of Natural Sciences, Technical University of Munich James-Franck-Straße 1 85748 Garching Germany; f Laboratory for Neutron Scattering and Imaging, Paul Scherrer Institut Forschungsstraße 111 5232 Villigen Switzerland; g Soft Matter and Functional Materials, Helmholtz-Zentrum für Materialien und Energie GmbH Hahn-Meitner-Platz 1 14109 Berlin Germany; h Max Planck Institute for Chemical Physics of Solids Nöthnitzer Straße 40 01187 Dresden Germany

## Abstract

The growth of single crystals of FeCl_3_, through sublimation and from the melt, is presented alongside a thorough investigation of their magnetostructural properties through a combination of DC magnetization and AC magnetic susceptibility measurements, single crystal X-ray diffraction (SCXRD), neutron powder diffraction (NPD) and small-angle neutron scattering (SANS). A new chiral polymorph of FeCl_3_ is identified, crystallizing in the non-centrosymmetric space group *P*3_1_. NPD and SANS reveal that a weakly first-order magnetic phase transition occurs from a paramagnetic phase with significant short-range correlations to an antiferromagnetic phase at *T*_N_ = 8.6 K, best described by the magnetic propagation vector ***k*** = (1/2, 0, 1/3) which differs from the previously reported magnetic structure of the well-known centrosymmetric polymorph (space group *R*3̄). We show that disordered crystallographic models including a large number of stacking faults are required to accurately reproduce the scattering observed in NPD patterns, preventing full determination of the magnetic structure. The magnetic field and temperature-dependent behavior of the intensities of the ***k*** = (1/2, 0, 2/3) and (1/2, 0, 5/3) magnetic Bragg peaks measured by SANS suggest that a field-induced spin reorientation occurs at *H* = 40 kOe when ***H***‖*c*-axis and at a significantly lower field of *H* ≈ 25 kOe when ***H***⊥*c*-axis. Above these magnetic fields in both cases the spins lie predominantly in the basal plane. The long-range magnetic ordering and the field-induced transitions observed in the neutron scattering experiments coincide with anomalies observed in the magnetisation *versus* both temperature and applied field along the principal crystal directions.

## Abstract 2

Single crystals of FeCl_3_ are grown from the melt and *via* sublimation in a new structural polymorph with chiral *P*3_1_ symmetry. The magnetic structure is investigated using neutron powder diffraction and small-angle neutron scattering.

## Graphical abstract

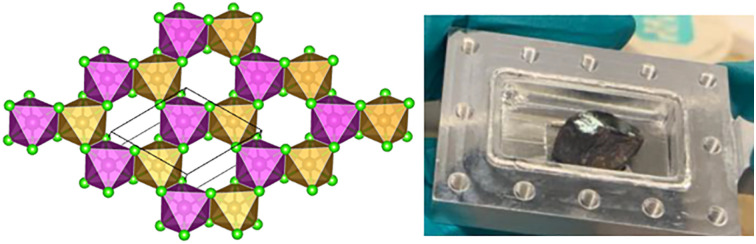

## Introduction

Magnetic frustration effects can lead to the formation of exotic magnetic phases. In magnetically frustrated systems, ordered non-colinear structures such as helimagnetic^1–4^ or magnetic skyrmion phases can be stabilized.^5–8^ On the other hand, disordered magnetic phases such as spin glasses,^9^ spiral spin liquids^10,11^ and spin ices^12^ can also arise. FeCl_3_, a van der Waals magnet, is an example of such a system where the combination of competing magnetic interactions that result from its layered honeycomb structure leads to a helimagnetic ground state^11,13,14^ and a rare spiral spin liquid phase above *T*_N_.^11^ van der Waals magnets in general have become of great interest since the observation of Ising-like out-of-plane ferromagnetism in atomically thin samples of CrI_3_^15^ and other two-dimensional materials.^16–19^

The magnetic ground state of frustrated systems is known to be sensitive to small perturbations^20,21^ and can therefore exhibit strong coupling with the underlying nuclear structure^22^ and structural defects such as stacking faults.^23–26^ Consistent with this notion, it was recently reported that the magnetic properties of FeCl_3_ are highly sensitive to small changes in the halide content when Cl is partially substituted by Br.^27^ Additionally, upon intercalation with one or two layers of graphite in the van der Waals gap, the magnetic properties of FeCl_3_ are severely altered and both long-range periodically ordered and spin-glass phases are observed.^28,29^

The existing literature on FeCl_3_ reports a variety of different magnetic properties. Initial neutron diffraction studies showed that helimagnetic order sets in at *T*_N_ = 15 K, below which the magnetic structure can be described by a spin spiral propagating along the hexagonal [145̄0] direction with a propagation vector (*k*-vector) of ***k*** = (4/15, 1/15, 3/2), where the spins in adjacent planes are rotated by 2π/15.^13^ Along the *c*-axis, the spins were found to be coupled antiferromagnetically.^13^ Subsequent neutron diffraction studies suggested that *T*_N_ ≈ 8 K and that the magnetic phase transition is weakly of first-order.^14^ The magnetic propagation vector was found to vary continuously with temperature.^14^ In both sets of neutron diffraction data, significant diffuse magnetic scattering was observed at temperatures *T* ≫ *T*_N_.^13,14^ A more recent neutron scattering study performed in 2022 revealed rings of diffuse scattering, consistent with a spiral spin liquid phase, that appear above *T*_N_ and persist up to *T* ∼ 20 K. Below *T*_N_, the same helimagnetic phase as earlier reported with ***k*** = (4/15, 1/15, 3/2) was found.

Temperature-dependent magnetization measurements, *M*(*T*), performed by Jones *et al.* on polycrystalline FeCl_3_ showed a single ordering peak at *T* = 9.75 K.^30^ In contrast, Bizette *et al.* observed two peaks in *M*(*T*) measurements on single crystals at *T* = 25 K and 10 K, in a magnetic field of *H* < 18 kOe (***H***‖*c*-axis).^31^ Additionally, the same authors performed isothermal magnetic-field-dependent magnetization measurements, *M*(*H*), and observed a field-induced transition at *H* = 15 kOe (***H***‖*c*-axis) below *T* = 20 K,^31^*i.e.* well above *T*_N_. Mössbauer spectra collected in applied magnetic fields below *T*_N_ suggested that the original spiral spin structure is absent for *H* > 15 kOe and instead two distinct hyperfine fields were observed which were fitted using a two-sublattice model.^32^ In this model the spins point along the Fe–Cl bonds. A second field-induced transition was observed for *H* = 40 kOe into a spin-flop-like phase where the spins are reoriented perpendicular to the applied field and lie in the basal plane.^32^ This field-induced transition has also been observed by AC susceptibility measurements.^33^ On the other hand, NMR experiments performed as a function of applied magnetic field and temperature on polycrystalline samples showed a continuous reorientation of the spins perpendicular to the applied field up to *H* = 40 kOe and found no evidence for the previously reported field-induced transition at *H* = 15 kOe.^34^

From the brief literature review presented above it is clear that several key open questions remain regarding the magnetic properties of FeCl_3_. The conflicting reports might be due to the existence of several different polymorphs of FeCl_3_; although the crystal structure is generally considered to adopt rhombohedral *R*3̄ symmetry with an ABC-stacking sequence of the honeycomb layers,^35–37^ two other polymorphs have been reported with space groups *P*312 and *P*3̄.^37^ These structures exhibit stacking of type ABB- and ABACBACBACBC- respectively, and their magnetic properties were not investigated. We note that *R*3̄ symmetry is confirmed experimentally in only one of the studies of magnetic properties discussed above;^11^ the rest either assume that the samples adopt *R*3̄ symmetry or do not mention details of the structure. In the current work we synthesize a novel non-centrosymmetric polymorph of FeCl_3_, crystallizing in the space group *P*3_1_, and probe the temperature and field dependence of the magnetization and magnetic structure using a combination of DC magnetization and AC susceptibility measurements, neutron powder diffraction (NPD) and small angle neutron scattering (SANS) experiments. We show that the magnetic structure is different to that of the *R*3̄ phase.

## Results

### Single crystal X-ray diffraction

**Fig. 1:**
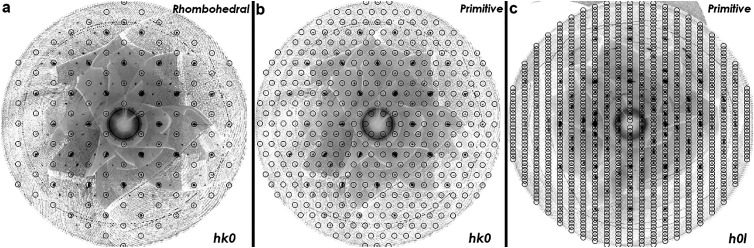
(a) and (b) *hk*0 and (c) *h*0*l* precession images constructed from single crystal X-ray diffraction data with overlaying circles indicating the position of allowed diffraction spots according to rhombohedral and primitive symmetry.

The unit cell parameters of a crystal grown by method **3** were determined to be *a* = *b* = 6.056(15) Å and *c* = 17.354(45) Å (*α* = *β* = 90° and *γ* = 120°), corresponding to either a primitive hexagonal or rhombohedral (hexagonal setting) cell. The construction of precession images from the raw data frames allows for the inspection of systematic absences due to lattice centring or certain symmetry elements. Open circles indicating the position of allowed reflections for rhombohedral and primitive lattices are superimposed on the *hk*0 and *h*0*l* precession images in Fig. 1. The rhombohedral *R* centering (obverse setting) imposes the reflection condition −*h* + *k* + *l* = 3*n* on the system. It is clear from Fig. 1(a) that this reflection condition is not obeyed and that additional diffraction spots are observed which can only be indexed using a primitive hexagonal cell as shown in Fig. 1(b). The *h*0*l* precession image shown in Fig. 1(c) reveals that the 00*l* = 3*n* reflection condition is obeyed, implying the presence of a threefold screw axis along the *c*-axis. These observations also hold in the case of the single crystals grown by method **1**. The observed reflection conditions limit the possible space groups describing the structure to the following chiral space groups: *P*3_1_, *P*3_2_, *P*3_1_12, *P*3_1_21, *P*3_2_12 and *P*3_2_21. The best fit was achieved using an almost racemic mixture of space group *P*3_1_ and its inversion twin *P*3_2_ (ratio *P*3_1_/*P*3_2_ = 0.4(3)). This *P*3_1_ polymorph of FeCl_3_ has hitherto not been observed in the literature. We note that Hashimoto *et al.* observed weak extra reflections obeying the condition *h* − *k* + *l* = 3*n* in a dedicated structural investigation of FeCl_3_ crystals, and ascribed these to the presence of a minor twin domain with an ACB- stacking sequence but retaining *R*3̄ symmetry.^36^ In our study all possible reflections are present in the *hk*0 plane (Fig. 1(a) and (b)), which cannot be accounted for by such twinning within a rhombohedral structure. The refinement parameters, fractional coordinates, equivalent isotropic and anisotropic displacement parameters, bond lengths and bond angles associated with the *P*3_1_ structure are shown in the ESI† in Tables S1–S5. The statistical fit factors are relatively high due to the smeared and broad nature of a significant fraction of the reflections, which is particularly apparent in Fig. 1(c). van der Waals materials are prone to stacking faults, which can lead to such smeared reflections. The layered crystal structure of the *P*3_1_ polymorph of FeCl_3_ viewed along the *a*-axis is shown in Fig. S1(a) (ESI†) and the in-plane honeycomb lattice is shown in Fig. S1(b) (ESI†).

The *P*3_1_ polymorph also exhibits ABC-stacking and differs only from the *R*3̄ polymorph in the octahedral coordination of the iron atoms. Shown schematically in Fig. S1(c) (ESI†), in the *P*3_1_ polymorph there are two distinct crystallographic Fe sites, both of which are 3a Wyckoff sites, and there are three unique Fe–Cl bond distances associated with each of the two FeCl_6_ octahedra, ranging from 2.305(17) Å to 2.434(15) Å. In the *R*3̄ polymorph there is only one crystallographic Fe site (6c Wyckoff position) with two very similar Fe–Cl bond distances (2.3734(9) Å and 2.3771(9) Å).^37^ Although we are unable to give a definitive explanation of why the *P*3_1_ polymorph of FeCl_3_ is formed, we speculate that it might originate from a small number of Fe or Cl vacancies that are partially ordered on one of the two octahedra.

### Magnetometry

**Fig. 2:**
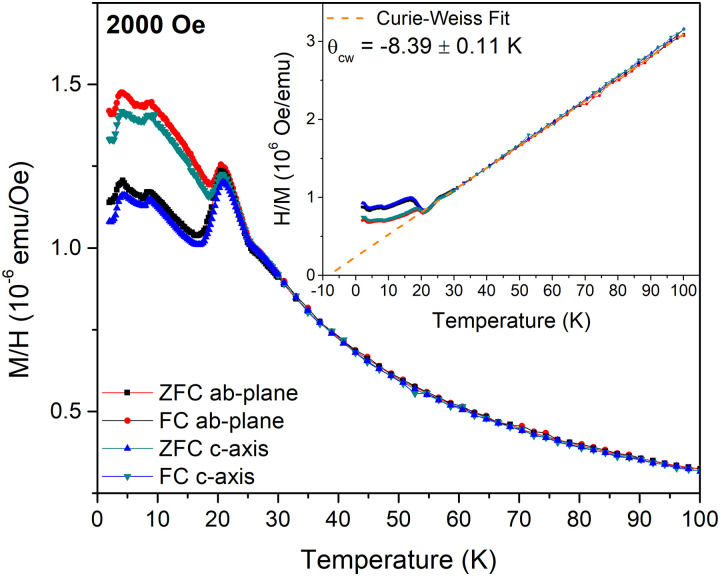
Magnetic field normalized zero-field-cooled (ZFC) and field-cooled (FC) magnetization, *M*/*H*, measured along the *c*-axis or the *ab*-plane and plotted against temperature. The inset shows the FC and ZFC inverse field normalized magnetization, *H*/*M*, plotted *versus* temperature. The dashed line indicates the Curie–Weiss law fit to the data for *T* > 30 K.

The magnetic properties of FeCl_3_ were investigated using temperature and field-dependent magnetization and AC magnetic susceptibility measurements. The temperature-dependent zero-field-cooled (ZFC) and field-cooled (FC) field-normalized magnetization, *M*/*H*, measured along the *ab*-plane and *c*-axis under an applied magnetic field of *H* = 2000 Oe, is shown in Fig. 2. Three peaks are observed at *T* = 20.75 K, 8.5 K and 4.25 K for all measurement protocols. Furthermore, below the onset of the peak at *T* = 20.75 K, a splitting is observed between the ZFC and FC data suggesting the onset of a dynamic magnetic process. Below *T* = 20.75 K the magnetization measured along the two principal directions exhibits weak anisotropy as *M*_c_/*M*_ab_ = 0.98. The temperature dependence of *H*/*M*, where *M* is the magnetization under an applied field of *H* = 2000 Oe, is shown in the inset of Fig. 2. Above *T* = 25 K, *H*/*M* increases linearly with temperature as expected for a paramagnet. A fit of the data to a Curie–Weiss law yields a Curie–Weiss temperature of *θ*_cw_ = −8.39 ± 0.11 K, indicating that antiferromagnetic interactions play a dominant role in this system. Large uncertainties with regard to the sample mass exist due to the difficulties related to weighing the small samples inside the glove box. The application of paraffin wax on the sample surface to prevent oxidation also prevented accurate determination of the mass outside the glove box, resulting in an inability to accurately determine the effective moment.

The real, *χ*′(*T*), and imaginary, *χ*′′(*T*), parts of the AC susceptibility are shown in Fig. S2(a) and (b) (ESI†) respectively. *χ*′(*T*) exhibits three peaks at similar temperatures to those observed in the *M*/*H*(*T*) curves shown in Fig. 2. No significant change of *χ*′(*T*) is observed by varying the frequency of the AC magnetic field. Consequently, *χ*′′(*T*) is almost zero and without any anomalies.

**Fig. 3:**
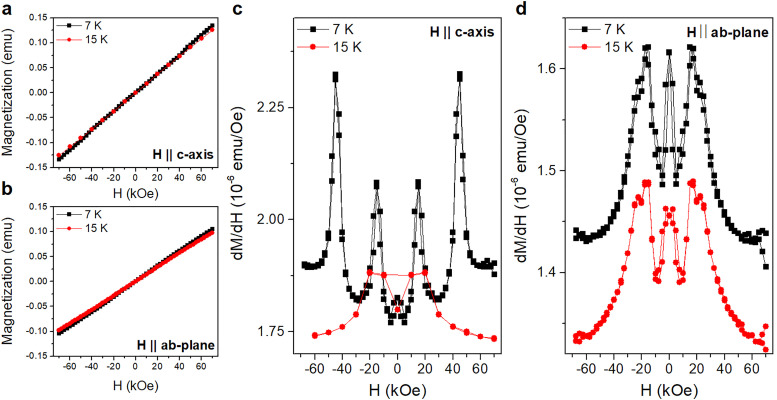
(a) and (b) Magnetization *versus* applied magnetic field and (c)and (d) its numerical derivative d*M*/d*H* measured along the *c*-axis and *ab*-plane at 7 K and 15 K.

The magnetic-field-dependent magnetization, *M*(*H*), measured along the *c*-axis and the *ab*-plane at *T* = 7 K and 15 K, corresponding to temperatures above and below the transition at 9 K, is shown in Fig. 3 alongside its numerical derivative d*M*/d*H*. As shown in Fig. 3(a) and (b), the *M*(*H*) curves are roughly linear but with small deviations from linearity that are most visible in the d*M*/d*H*(*H*) curves (Fig. 3(c) and (d)). At *T* = 7 K and along the *c*-axis, d*M*/d*H* reveals maxima at |*H*| = 15 kOe and 40 kOe, with the latter vanishing upon increasing the temperature to *T* = 15 K. On the other hand, the *M*(*H*) curves measured along the *ab*-plane exhibit a slightly S-shaped character for both temperatures. The corresponding d*M*/d*H* curves shown in Fig. 3(d) show an initial decrease above/below 0 kOe and a peak centered at |*H*| = 15 kOe alongside a shoulder at |*H*| = 22.5 kOe. These features are prominent at *T* = 7 K but persist, although weaker, when the temperature increases to *T* = 15 K.

The peaks of the d*M*/d*H*(*H*) curves along the *c*-axis have previously been reported in the literature.^31,32^ However, to our knowledge the transitions observed in the d*M*/d*H*(*H*) curves measured with a field applied in the *ab*-plane have not been reported in the literature thus far. The similarity in the value of the critical field for the |*H*| = 15 kOe out-of-plane transition and the weak magnetic anisotropy of this system suggest that the same process could plausibly be responsible for the peaks along both orientations. The application of a magnetic field in the basal plane could also shift the critical field required to achieve the spin-flop-like phase (in which the spins lie in the basal plane) to lower fields, possibly accounting for the shoulder at *H* = 22.5 kOe in Fig. 3(d).

### Neutron powder diffraction

**Fig. 4:**
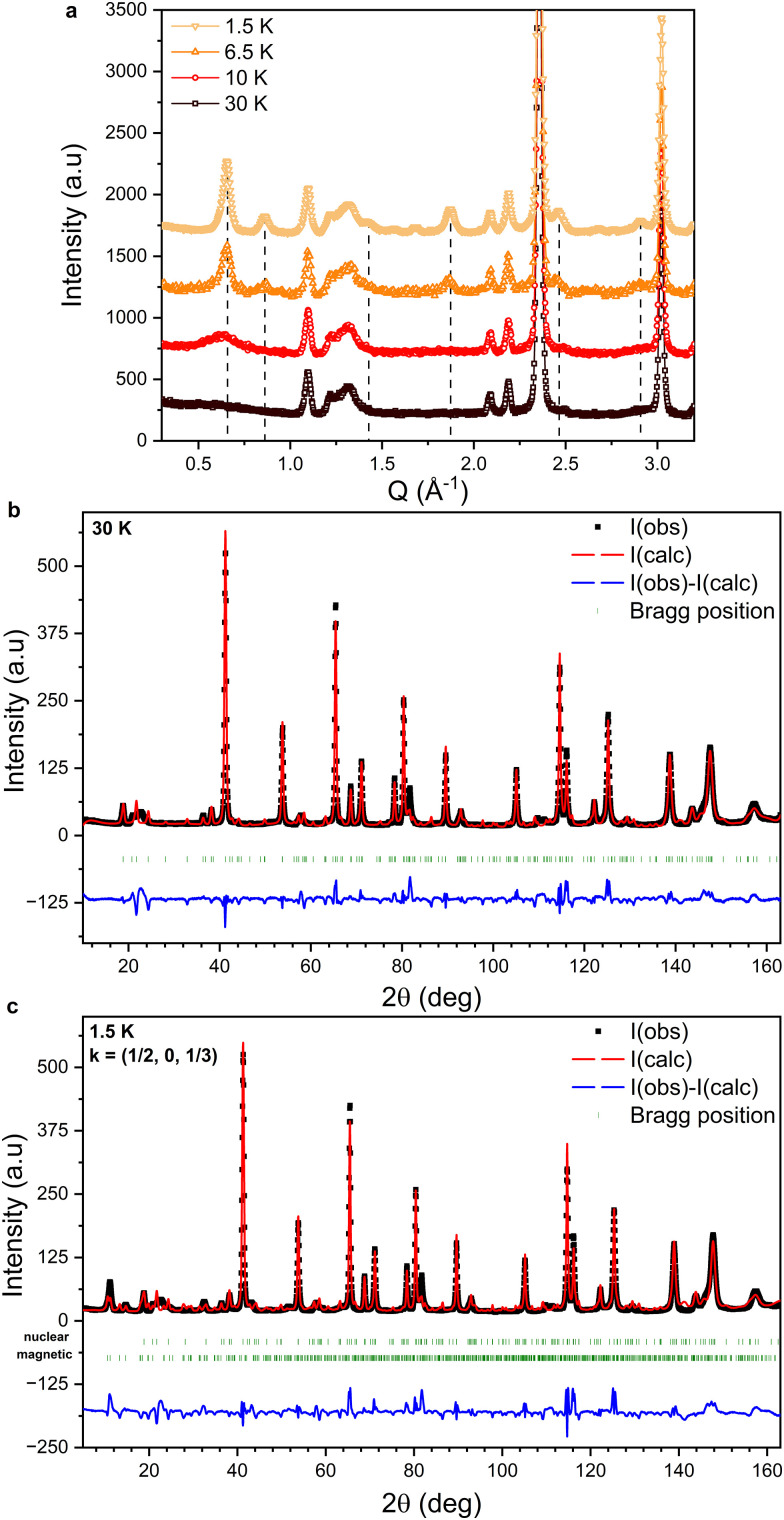
(a) Evolution of the low-angle part of the NPD patterns collected between 30 K and 1.5 K. The dashed lines indicate the positions of magnetic peaks. The data are plotted with a fixed offset for clarity. (b) NPD data collected at 30 K fitted using the nuclear structure of FeCl_3_ (*P*3_1_). (c) NPD data collected at 1.5 K fitted using the nuclear (*P*3_1_) and magnetic (*P*_*S*_1) structures of FeCl_3_. The green markers indicate the expected peak positions for the nuclear (upper) and magnetic (lower) structures. The difference between *I*_(calc)_ and *I*_(obs)_ for both refinements is plotted in blue.

Neutron powder diffraction measurements were performed at various temperatures between *T* = 200 and 1.5 K. The effect of lowering the temperature from *T* = 30 K to 1.5 K on the low-angle part of the diffraction pattern is shown in Fig. 4(a). At *T* = 30 K, peaks corresponding only to the nuclear structure are observed. At *T* = 10 K, slightly above the transition observed at *T* = 8.75 K in the *M*(*T*) curves shown in Fig. 2, a broad diffuse magnetic scattering peak is observed at *Q* = 0.65 Å^−1^, indicative of short-range magnetic correlations. Below *T* = 8.75 K, several sharp magnetic peaks are observed (indicated by the dashed lines in Fig. 4(a)), providing evidence for a long range ordered magnetic phase.

No additional intensity is observed at the nuclear peak positions, confirming the antiferromagnetic nature of the low temperature phase. We therefore deduce that *T*_N_ = 8.75 K in accordance with previous reports.^14,32,33^ However, the magnetic structure reported by Cable *et al.*^13^ would have given rise to a zeroth order satellite peak, corresponding to the pitch of the helix at *Q* = 0.367 Å^−1^. It is clear from Fig. 4(a) that no magnetic scattering is observed below *Q* = 0.5 Å^−1^. Therefore, our results indicate the stabilization of a different magnetic structure than that reported by Cable *et al.*^13^

### *k*-Vector and symmetry analysis

The *k*-vector was determined using the K-search program as implemented in the Fullprof software suite^38^ using the *P*3_1_ phase described above as the paramagnetic parent phase (*P*3_1_1′ paramagnetic space group). We found that ***k*** = (1/2, 0, 1/3) gives the best agreement with the magnetic peak positions. Notably, this is different from the previously reported value of ***k*** = (4/15, 1/15, 3/2).^11,13,14^ The ISODISTORT routine from the ISOTROPY software suite^39,40^ and the k-Subgroupsmag program from the Bilbao crystallographic server^41^ were used to explore the effect of the determined vector ***k*** = (1/2, 0, 1/3) on the symmetry of *P*3_1_1′. Two possible magnetic space groups are identified, *P*1 (#1.1) and *P*_*S*_1 (#1.3), depending on how many arms of the star of ***k*** are active. The magnetic space group *P*_*S*_1 is stabilized in the case of one or two active *k*-vectors while the magnetic space group *P*1 is stabilized in the case of three active *k*-vectors. In *P*_*S*_1, the spins are coupled antiferromagnetically along the in-plane directions. The magnetic order along the *c*-axis is rather poorly defined, possibly implying more complex ordering such as sinusoidal modulation. Ferromagnetic ordering is forbidden by symmetry for the magnetic space group *P*_*S*_1, while it is not forbidden in the magnetic space group *P*1. Furthermore, the magnetic phase transition from the paramagnetic space group *P*3_1_1′ (*G*_0_) to the ordered phase described by space group *P*_*S*_1 (*G*_*k*_) is forbidden to be second-order as the index of the subgroup is |*G*_0_|/|*G*_*k*_| = 3,^42,43^ meaning the number of symmetry operations is decreased by a factor of three as a result of the transition.

Representational analysis was performed using the BasIReps program^44^ as implemented in the Fullprof software suite. A single irreducible representation is found to correspond to the magnetic structure (*P*_*S*_1) described by the paramagnetic space group and the *k*-vector described above. For the two Fe-sites, three real basis functions along the *a*-, *b*- and *c*-axis describe the magnetic moments and the corresponding coefficients are determined during the Rietveld refinement.

We note that the *k*-vector of (1/2, 0, 1/3) is common to different samples. Fig. S4 of the ESI,† compares NPD patterns collected at *T* = 2 K (PEARL diffractometer at TU Delft) on the current sample prepared by sublimation in an evacuated ampoule, and on a second sample purified using chemical vapor transport in an ampoule containing Cl_2_ gas (see Experimental methods). The magnetic peaks for the two samples are located at the same positions.

### Refinement

The experimental diffraction pattern collected at *T* = 30 K, which only contains reflections from the nuclear structure, is shown in Fig. 4(b) alongside the fitted intensity from Rietveld refinement. The structure was refined in the space group *P*3_1_ and the cell parameters are *a* = *b* = 6.04457(6) Å and *c* = 17.2937(4) Å. Preferred orientation effects due to the stacking of plate-like crystallites in the sample container were modelled by the refinement of spherical harmonic coefficients. Anisotropic size broadening effects were also taken into account. Although most peaks are well fitted, the calculated pattern contains a number of low intensity peaks distributed across the entire 2*θ* range that are not observed experimentally. Furthermore, a broad feature is observed at 2*θ* ≈ 22° that is not reproduced by the structural model; this is discussed in detail below.

The diffraction pattern collected at *T* = 1.5 K, *i.e.* well below *T*_N_, is shown in Fig. 4(c), fitted by performing a Rietveld refinement of the nuclear and magnetic structures. Although there is good agreement between the calculated and observed magnetic peak positions, the fit to the experimental data suffers from an inadequate description of the nuclear structure, leading to large unphysical values for the refined magnetic moments of the Fe atoms. This prevents a precise determination of the magnetic structure.

### Stacking faults

Broadened diffraction peaks and the suppression of low intensity reflections such as those observed in Fig. 4 are commonly found for layered systems with large concentrations of planar defects such as stacking faults. Modeling such defects and their diffraction characteristics requires an alternative to the standard Rietveld refinement method attempted above. The FAULTS program^45^ as implemented in the Fullprof software suite can be used to simulate diffraction patterns for materials with varying ratios of stacking faults and to refine stacking fault models against the experimental data.

**Fig. 5:**
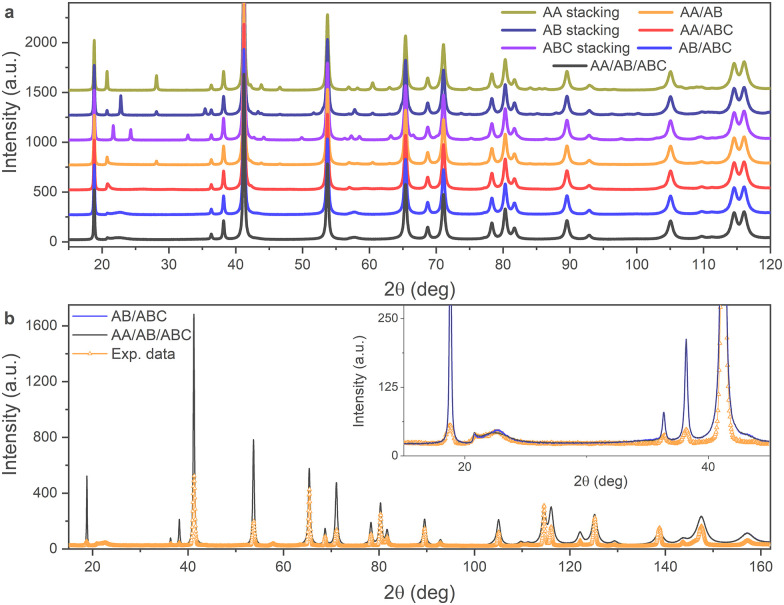
(a) Simulated diffraction patterns for several faulted models of FeCl_3_. (b) Simulated diffraction patterns for AB/ABC and AA/AB/ABC faulted models of FeCl_3_.

The structural model used for the FAULTS program is not a periodic three-dimensional structure but is built up from separately defined layers that are related to each other through transition vectors (***t***_***ij***_, where *i* and *j* represent the transition from layer *i* to layer *j*) which have an associated transition probability (*α*_*ij*_). Here we use a model consisting of three identical FeCl_3_ layers (with Laue symmetry −3) with transition vectors ***t***_11_ = ***t***_22_ = ***t***_33_ = (0, 0, 1), ***t***_12_ = ***t***_23_ = ***t***_31_ = (2/3, 1/3, 1) and ***t***_13_ = ***t***_21_ = ***t***_32_ = (−2/3, −1/3, 1). Depending on the values of *α*_*ij*_, non-faulted stacking sequences as well as stacking fault (SF) phases can be modeled. In Fig. 5(a) simulated diffraction patterns are shown for a pure AA stacking phase, pure AB stacking phase, pure ABC stacking phase, three SF phases with two possible ***t***_***ij***_ where the corresponding *α*_*ij*_ values are each set to 0.5 (AA/AB, AA/ABC and AB/ABC) and one SF phase where all three ***t***_***ij***_ are possible and where all *α*_*ij*_ = 1/3. The patterns were simulated using the profile parameters obtained through the Rietveld refinements described above. Note that the scale of the simulated intensity is chosen manually such that intensities of the experimental and simulated diffraction patterns are comparable. Large differences between the models are observed up to 2*θ* ≈ 80°. The broad peak observed experimentally at 2*θ* = 22° is reproduced in the case of the AB/ABC and AA/AB/ABC stacking fault models. The sharp diffraction peaks associated with the ordered stacking sequences below 2*θ* = 40° are suppressed in all the stacking fault models except for the AA/AB model, illustrating the necessity of including ABC stacking. The simulated diffraction patterns of the AB/ABC and AA/AB/ABC phases are compared to the experimental data in Fig. 5(b). Good agreement between the simulated and experimental data is found. The differences between the two stacking fault models shown in Fig. 5(b) are minor. We note that the most intense diffraction peaks are not modeled accurately, which is likely due to the inability to include preferred orientation effects in the stacking fault models.

Fig. S3 (ESI†) shows the experimental diffraction pattern collected at *T* = 30 K and the calculated pattern obtained from refinement of the stacking fault phase. As seen previously in the simulated data, the refined pattern reproduces the broad feature at 2*θ* = 22°, although the magnitude of the intensity is not matched fully, and low intensity reflections are suppressed over the entire 2*θ* range. The transition probabilities for the transition vectors refined to *α*_11_ = *α*_22_ = *α*_33_ = 0.127, *α*_12_ = *α*_23_= *α*_31_ = 0.396 and *α*_13_ = *α*_21_ = *α*_32_ = 0.478. The inability to model preferred orientation effects in the FAULTS program leads to unreliable values for the scale factor, which leads to the underfitting of several high intensity peaks and more importantly leads to large uncertainties for the refined values of *α*_*ij*_. We also observed that the refined values of *α*_*ij*_ depend to some extent on their initial values, which reflects the influence of local minima during the minimization procedure. Although accurate values for *α*_*ij*_ cannot be obtained, it is apparent from the simulated data that stacking faults are essential in order to reproduce the features of the experimental diffraction pattern and that the best fit is obtained with primarily a mixture of ABC and AB stacking, with the possible presence also of AA stacking.

We note that the broad feature attributed to stacking faults is also observed in an NPD pattern of the second sample that was purified using chemical vapor transport in an ampoule containing Cl_2_ gas (Fig. S4 of the ESI†).

### Small angle neutron scattering

Temperature and magnetic-field-dependent small angle neutron scattering experiments were performed on single crystal samples on the V4 instrument at the Helmholtz Zentrum Berlin (HZB). The measurements were performed in transmission geometry and the direction of *H* was parallel to the incoming neutron beam propagation vector (***k***_***i***_). The rotation of the sample could be controlled during the experiment and the sample was oriented such that the rotation (vertical) axis lay in the *ab*-plane. The rotation angle is denoted as *ω*, and its value represents the angle between ***k***_***i***_ and the *c*-axis of the crystal structure.

**Fig. 6:**
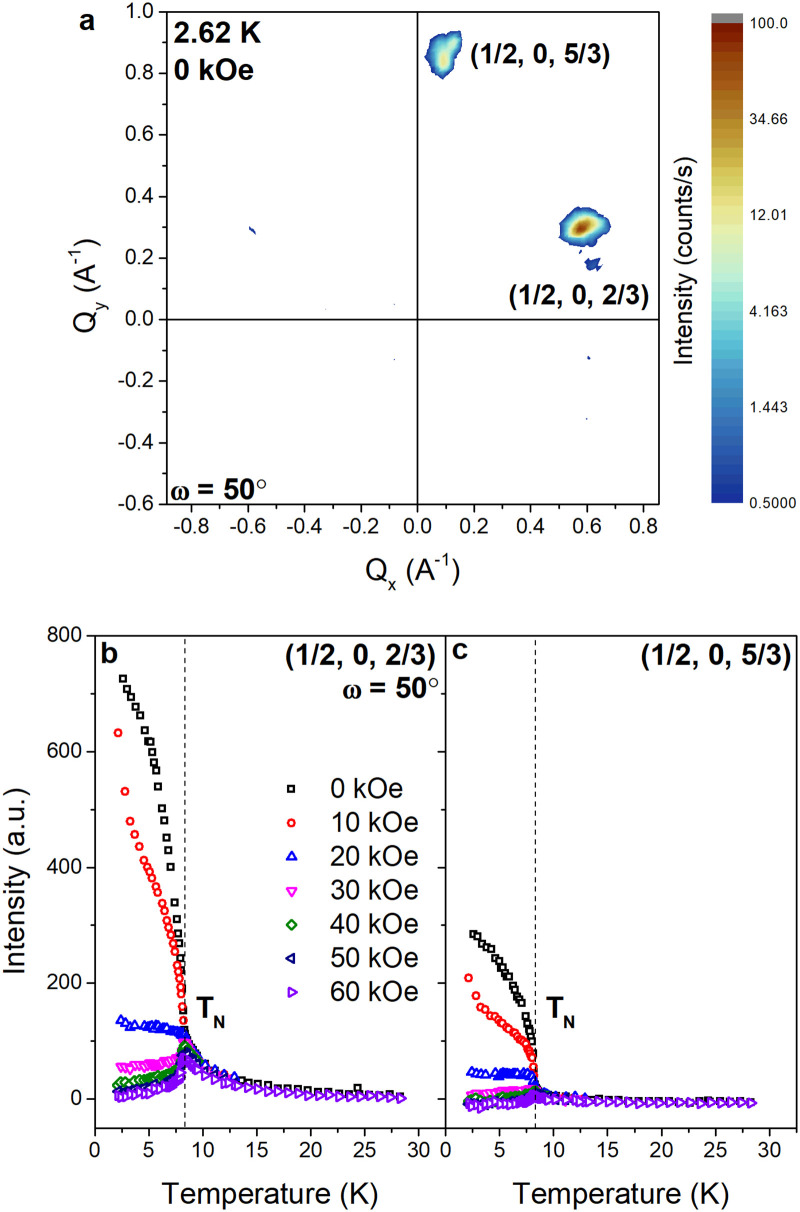
(a) SANS pattern collected in zero applied field at *T* = 2.62 K and *ω* = 50°. The ***k*** = (1/2, 0, 2/3) and (1/2, 0, 5/3) reflections are indicated. (b), (c) Integrated intensities of the ***k*** = (1/2, 0, 2/3) and (1/2, 0, 5/3) reflections plotted against temperature, collected under the applied magnetic fields indicated by the legend. The Néel temperature is indicated by the dashed line.

Rocking scans revealed the presence of two magnetic Bragg peaks below *T*_N_, centered at *Q* = 0.65 Å^−1^ and 0.86 Å^−1^. The two peaks are visible simultaneously at *ω* = 50° as shown in Fig. 6(a) and can be indexed as ***k*** = (1/2, 0, 2/3) and ***k*** = (1/2, 0, 5/3) respectively; they coincide with the first two magnetic peaks observed in the NPD patterns in Fig. 4(a), indicating that the same magnetic structure is stabilized in single crystalline samples as in powdered samples.

The temperature dependence of the integrated intensities, *I*(*T*), of the two peaks at *ω* = 50° under applied magnetic fields ranging from *H* = 0 to 60 kOe are shown in Fig. 6(b) and (c). In zero field, the intensity of both peaks increases sharply below *T* = 8.3 K and tends to saturation at lower temperatures, although full saturation is not achieved within the temperature range of our experiment. For *H* = 10 kOe, *I*(*T*) of both peaks follows a similar trend down to *T* = 4.1 K, below which the slope increases significantly. For *H* = 20 kOe, *I*(*T*) reaches a plateau below *T*_N_ and is significantly lower than in smaller applied fields. For *H* ≥ 30 kOe, the intensity reaches a maximum at *T*_N_ and then decreases with further decreasing temperature. For all applied fields, significant intensity is observed up to ∼2*T*_N_ particularly at the position of the ***k*** = (1/2, 0, 2/3) peak, implying the presence of short-range magnetic correlations that are also observed in the NPD patterns shown in Fig. 4(a) and have previously been reported in the literature.^11,13,14^

**Fig. 7:**
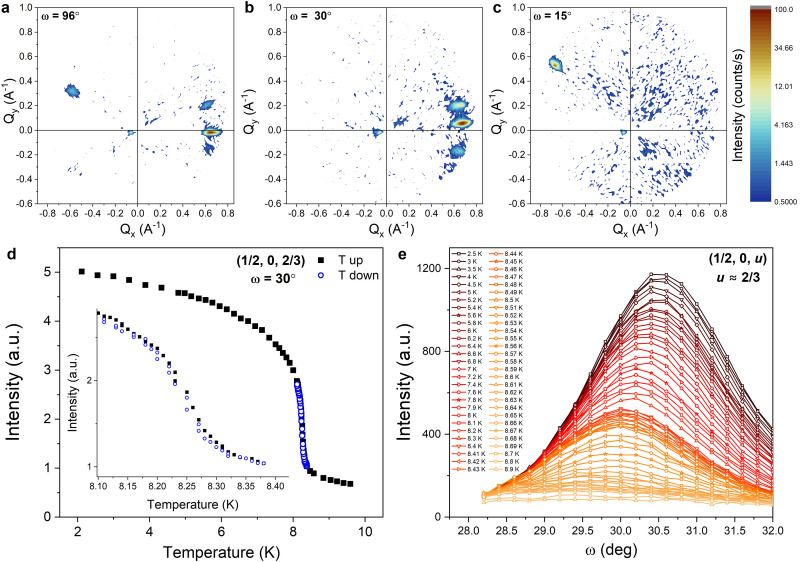
SANS patterns collected in zero applied field with different crystal orientations where (a) *ω* = 96°, (b) *ω* = 30° and (c) *ω* = 15°. (d) Temperature dependence of the integrated intensity of the ***k*** = (1/2, 0, 2/3) magnetic reflection upon warming and cooling through *T*_N_. (e) Rocking curve measurements showing a shift of the maximum of the ***k*** = (1/2, 0, *u*) magnetic reflection, where *u* ≈ 2/3, with varying temperature.

The anisotropic response of the magnetic intensity was investigated using three different orientations of the sample with respect to ***k***_***i***_ and is shown in Fig. 7(a)–(c). Fig. 7(a) and (b) also show the presence of multiple diffracting domains by the appearance of satellite reflections around the main (most intense) peak with identical values of *Q* but with intensities that vary with *ω*.

The intensity at the position of the ***k*** = (1/2, 0, 2/3) peak measured upon heating and cooling in the vicinity of *T*_N_ in zero field is shown in Fig. 7(d). Hysteretic behavior is observed with a small width on the order of 0.01 K, which is consistent with observations made by Endoh *et al.*^14^ and suggests that the transition is weakly of first-order as expected from the symmetry analysis. The inflection point of the temperature derivative of the intensity leads to *T*_N_ = 8.6 K.

The temperature dependence of the integrated intensity of the ***k*** = (1/2, 0, 2/3) peak below *T*_N_ in zero field, collected with rocking scans around *ω* = 30°, is shown in Fig. 7(e). The angle at which the peak exhibits a maximum shifts towards smaller angles with increasing temperature, from *ω* = 30.5° at *T* = 2.5 K to *ω* = 29.4° at *T* = 8.9 K. This temperature dependence of the maximum indicates that the propagation vector continuously changes below *T*_N_, which is consistent with the results of previous neutron scattering experiments [13]. As the *k*-vector determined above lies on the *U* symmetry line of the hexagonal Brillouin zone (***k*** = (1/2, 0, *u*)), it is plausible that the continuous change in the propagation vector reflects a continuous change in the value of *u*. The ***k*** = (1/2, 0, 2/3) reflection can therefore be indexed as ***k*** = (1/2, 0, *u*) where *u* = 2/3, as shown in Fig. 7(e). We note, however, that the continuous change of the propagation vector, and thereby its deviation from the commensurate value, cannot be discerned from the variable temperature NPD patterns in Fig. 4(a); the magnetic Bragg peak at *Q* = 0.65 Å^−1^ can thus be indexed as ***k*** = (1/2, 0, 2/3) within experimental error.

**Fig. 8:**
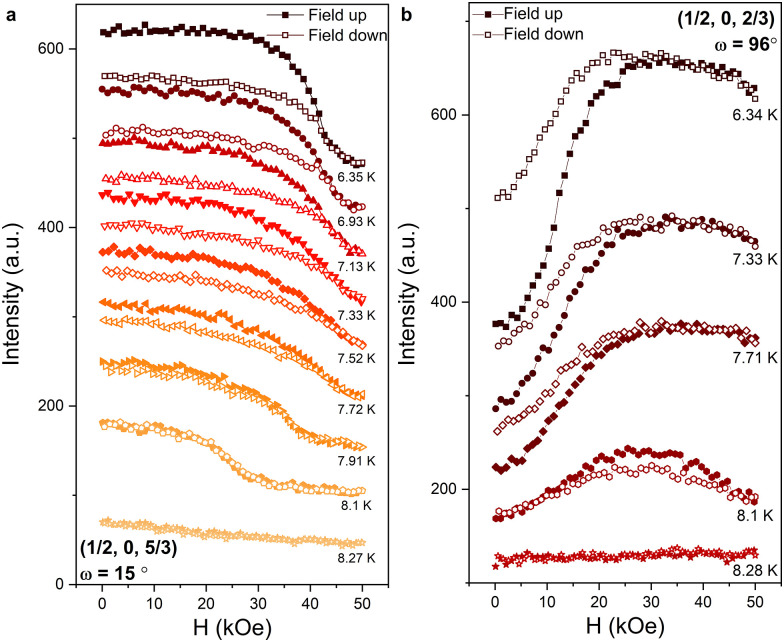
Hysteretic field-dependent intensity at the position of the (a) ***k*** = (1/2, 0, 5/3) and (b) ***k*** = (1/2, 0, 2/3) magnetic reflections around *T*_N_. The magnetic field is applied close to the *c*-axis and the *ab*-plane in (a) and (b) respectively.

The dependence of the integrated intensities of these two peaks (the main peaks at *ω* = 15° and 96° respectively) on cycling ***H*** from 0 to 50 kOe and back, in the vicinity of *T*_N_, is shown in Fig. 8(a) and (b). In Fig. 8(a), ***H*** is almost parallel to the *c*-axis and upon cooling below *T*_N_ the intensity of the (1/2, 0, 5/3) peak becomes strongly field dependent; a step from high to low intensity is observed at *H* ≈ 20 kOe. With decreasing temperature, the step in intensity shifts to higher fields, reaching ∼40 kOe at *T* = 6.35 K. The hysteresis associated with this process also becomes more pronounced with decreasing temperature. In Fig. 8(b), ***H*** is applied almost perpendicular to the *c*-axis and the intensity of the (1/2, 0, 2/3) peak follows a different trend. For *T* = 8.1 K, with increasing field the intensity increases and reaches a maximum between *H* = 20 and 30 kOe, above which it decreases. Upon cycling the field, a hysteresis loop is observed between *H* = 10 and 50 kOe. For *T* ≤ 7.71 K, the intensity increases up to *H* ≈ 25 kOe, above which it decreases slowly. Hysteresis is observed between *H* = 0 and 25 kOe upon cycling the field.

## Discussion

The neutron scattering data collected on polycrystalline and single crystal samples as well as the SCXRD experiments described in this work allow for a closer interpretation of the features observed *via* magnetometry. The SANS results show that the transition to a long-range magnetically ordered state occurs at *T*_N_ = 8.6 K, which is in agreement with the NPD and magnetometry results. Furthermore, this value for *T*_N_ is consistent with previous reports in the literature.^32–34,46^ Above this temperature short-range magnetic correlations are observed in the form of diffuse magnetic scattering persisting up to *T* ∼ 25 K.

Some features of the neutron powder diffraction data can only be explained when stacking faults are considered. This leads to the question of whether stacking faults influence the magnetic behavior of the samples. ABAB-type stacking faults in α-RuCl_3_ are known to lead to a different magnetic structure than that observed in pristine samples, significantly raising the ordering temperature as a consequence.^23,25,26^ A similar mechanism, in combination with the different crystallographic symmetry to previous reports of FeCl_3_, might give rise to the novel magnetic structure with ***k*** = (1/2, 0, 1/3) described here.

In mechanically deformed samples of α-RuCl_3_ a broad peak is observed in *M*(*T*) measurements above *T*_N_, comparable to the peak at *T* = 20.75 K observed in the current work on FeCl_3_. For α-RuCl_3_ the peak at *T* ∼ 20 K can occur together with another peak at the *T*_N_ of pristine samples.^23^ The diffuse scattering observed above *T*_N_ in both powder and single crystalline samples of FeCl_3_ suggests that local magnetic order or a spiral spin liquid phase^11^ is present. Furthermore, the anomaly at *T* = 20.75 K observed in our magnetometry results, which was also observed in *M*(*T*) measurements by Bizette *et al.*,^31^ cannot be attributed to the presence of magnetic impurity phases that would be expected if the FeCl_3_ sample was partially oxidized (such as FeOCl,^47^ FeOOH,^48,49^ FeO,^50^ Fe_2_O_3_ polymorphs^51^ and Fe_3_O_2_^51^) as their respective ordering temperatures do not coincide. We speculate that this feature might instead be a result of the stacking faults.

The origin of the peak in the *M*(*T*) measurements at *T* = 4.25 K is more difficult to ascertain. A change in slope of the magnetic peak intensities is observed at this temperature, as shown in Fig. 6(b) and (c). However, no change in the magnetic propagation vector is observed, and the origin of this feature will require further investigation.

The magnetic-field dependence of the ***k*** = (1/2, 0, 5/3) peak intensity, shown in Fig. 8(a), suggests that a reorientation of the spins occurs at *H* = 40 kOe when the magnetic field is applied parallel to the *c*-axis. This is consistent with the peak in the d*M*/d*H* curve shown in Fig. 3(c) at *H* = 40 kOe. As neutron scattering probes the magnetization component perpendicular to the scattering vector the decreasing intensity of the (1/2, 0, 5/3) peak with increasing *H* is consistent with a transition to a state where the spins lie predominantly in the basal plane, as previously concluded from a Mössbauer spectroscopy study.^32^

The magnetic-field dependence of the ***k*** = (1/2, 0, 2/3) peak intensity shown in Fig. 8(b) suggests that a reorientation of the spins also occurs at *H* ≈ 25 kOe when the field is applied perpendicular to the *c*-axis. This is also consistent with the peak in the d*M*/d*H* curve in Fig. 3(d) at *H* ≈ 25 kOe. The increasing intensity of the (1/2, 0, 2/3) peak with increasing *H* suggests again that the spins reorient towards the basal plane but at significantly lower fields than when the field is applied parallel to the *c*-axis.

No signature of a field-induced transition occurring at *H* = 15 kOe, as suggested by the peaks in the d*M*/d*H* curves both below and above *T*_N_ in Fig. 3(c), is observed in our neutron scattering data. The disappearance of the d*M*/d*H* peak at *H* = 40 kOe upon heating above *T*_N_, while the peak at *H* = 15 kOe remains visible, suggests that any transition occurring at *H* = 15 kOe is not associated with the long-range magnetic order stabilized below *T*_N_ but rather with the short ranged magnetic correlations that persist above *T*_N_. This is plausibly influenced by the stacking faults present in this system, akin to the effects that ABAB-type stacking faults have on the magnetic properties of RuCl_3_,^23,25,26^ or by the presence of a spiral spin liquid phase as reported for crystals of FeCl_3_ with the *R*3̄ structure.^11^

## Conclusions

In this work, we report on the synthesis of single crystals of FeCl_3_ by both sublimation and by using a custom-built Bridgman–Stockbarger furnace. Notably, the growth of centimeter sized crystals from the melt is possible using the latter method.

Single crystal X-ray diffraction showed that the crystals grown using both methods crystallize in the chiral space group *P*3_1_, which is a novel polymorph of FeCl_3_. Temperature-dependent NPD and SANS revealed a weakly first-order magnetic phase transition from a paramagnetic phase with significant short-range correlations to an antiferromagnetic phase at *T*_N_ = 8.6 K. The propagation vector best describing the magnetic structure was found to be ***k*** = (1/2, 0, 1/3), corresponding to a different magnetic structure than has previously been reported. The propagation vector was also found to be temperature dependent. Conventional Rietveld refinement of the NPD data collected at *T* = 30 K was found to be inadequate in fitting the experimental diffraction pattern due to the presence of complex peak broadening and diffuse scattering. These features could be reproduced by models comprising a large density of stacking faults. NPD patterns collected below *T*_N_ could therefore not be accurately fitted using any magnetic structural model.

The magnetic field and temperature-dependent behavior of the intensities of the ***k*** = (1/2, 0, 2/3) and (1/2, 0, 5/3) magnetic Bragg peaks measured in the SANS experiments suggest that a field-induced spin reorientation occurs at *H* = 40 kOe for ***H***‖*c*-axis and at significantly lower fields of *H* ≈ 25 kOe for ***H***⊥*c*-axis. Above these magnetic fields the spins lie predominantly in the basal plane.

The long-range magnetic ordering and the field-induced transitions observed in the neutron scattering experiments coincide with the anomalies observed in the *M*(*T*) and *M*(*H*) measurements along the principal directions of the structure. However, no evidence for the anomalies observed at *T* = 20 K in the *M*(*T*) curves and at *H* = 15 kOe (***H***‖*c*-axis) in the *M*(*H*) curves are observed in the neutron scattering experiments. The reproducibility of these anomalies together with previous reports of similar features in Mössbauer spectroscopy data and magnetization measurements suggest that they are of genuine nature and are potentially associated with the short-range magnetic correlations above *T*_N_, possibly due to the presence of stacking faults.

## Experimental methods

### Crystal growth

Single crystals of FeCl_3_ were grown by sublimation (method **1**) and by two different protocols (methods **2** and **3**) using a custom-built Bridgman–Stockbarger furnace. For the crystals grown by sublimation (method **1**), 3 g of commercially obtained FeCl_3_ powder of nominal 98% purity (Thermo Fisher Scientific) was loaded into a borosilicate glass ampoule (*L* = 30 cm and *ϕ*_out_ = 3 cm) in a nitrogen-filled glove box. ICP-MS analysis determined the purity to be higher (99.6%) than the nominal value, with the following impurities detected: Mn (0.25%), Cu (0.13%), Ni (0.02%). The ampoule was flame-dried before the addition of the FeCl_3_ powder to drive off any adsorbed water. The open end of an ampoule was connected to a Quickfit stopcock adapter, which was closed before removal from the glovebox. To seal the ampoule, the other end of the stopcock adapter was attached to a Schlenk line that was connected to a vacuum pump, the stopcock was opened, and the ampoule was evacuated to an approximate residual nitrogen pressure of 1 mbar. The ampoule was then flame sealed while the stopcock was in the open position. The sealed ampoule was placed in a three-zone furnace with a set temperature gradient of *T* = 290–305–320 °C and was kept under this temperature gradient for 15 h after which it was cooled down to room temperature over 10 h. Millimeter sized crystals grew in a clustered fashion at the cold side and were removed and mechanically isolated from the ampoule in a nitrogen filled glove box. During the initial crystal growth, reddish crystals were observed to grow approximately 1 cm below the main boule of FeCl_3_ crystals. Single crystal X-ray diffraction identified these crystals as FeOCl. To prevent the inclusion of such oxide impurity phases, the FeCl_3_ crystals were recrystallized twice following method **1**, after which no further FeOCl impurity crystals were observed. The resulting FeCl_3_ crystals were used in the diffraction and magnetometry experiments.

A second polycrystalline sample was prepared in a different laboratory using a modification of method **1** that differed from the procedure above in the following way. The initial FeCl_3_ powder, also of nominal 98% purity (Alfa Aesar), was purified by means of chemical vapor transport within a quartz ampoule in the presence of a Cl_2_ atmosphere.

For methods **2** and **3**, a custom Bridgman–Stockbarger furnace, shown in Fig. S5(e) (ESI†), was constructed from three hollow borosilicate cylinders (*L* = 60 cm) inserted into one another. The inner two cylinders are wrapped with Kantal FeCrAl alloy wire functioning as resistive heating elements. The outer and inner diameters of the three cylinders are: *ϕ*_out_1__ = 5.4 cm, *ϕ*_in_1__ = 4.9 cm, *ϕ*_out_2__ = 4.3 cm, *ϕ*_in_2__ = 3.9 cm, *ϕ*_out_3__ = 3.6 cm and *ϕ*_in_3__ = 3.15 cm. Over the length of the cylinders the spacing between the wrapped wire is gradually increased, resulting in a smooth temperature gradient along the furnace. The heating elements are controlled by a custom-built temperature controller connected to a type-K thermocouple positioned halfway along the furnace in the central cylinder. For the crystals grown in the custom-built Bridgman–Stockbarger furnace, ∼3.31 g of nominally 98% pure FeCl_3_ powder (Thermo Fisher Scientific) was initially loaded in a quartz ampoule (*L* = 12.5 cm and *ϕ*_out_ = 1.6 cm) in a nitrogen-filled glove box. The ampoule was flame-dried before addition of the FeCl_3_ powder to drive off any adsorbed water. The quartz ampoule was sealed following the same protocol described for method **1**. The sealed ampoule was then attached to a stepper motor by a wire and the power to the furnace was increased until *T* = 360 °C was reached. Two different crystal growth protocols were followed. In the first growth protocol (method **2**) the furnace was oriented such that the high temperature side was at the bottom and the ampoule was raised from the high to low temperature side of the furnace at a rate of 2 mm h^−1^, reaching completion after 14 days. This inverse Bridgman procedure resulted in crystal growth by sublimation. A large single crystal with clear hexagonal facets (6.7 × 6.7 × 11.2 mm), shown in Fig. S5(a)–(c) (ESI†), formed at the top of the ampoule. The crystal was removed and mechanically isolated from the ampoule in a nitrogen-filled glove box. In the second growth protocol (method **3**) the furnace was oriented such that the high temperature side was at the top and the ampoule was lowered from the high to low temperature side of the furnace at a rate of 6 mm h^−1^, reaching completion after 2 days. This procedure allowed crystal growth from the melt. Once the growth was completed, the crystals were removed and mechanically isolated in a nitrogen-filled glove box. Long crystals (average dimensions of 4 × 6 × 17 mm) were obtained, an example of which is shown in Fig. S5(d) (ESI†).

### Single crystal X-ray diffraction

SCXRD measurements were performed on FeCl_3_ single crystals grown by methods **1** and **3** using a Bruker D8 Venture diffractometer equipped with a Photon 100 CMOS detector, operating with Mo Kα radiation at *T* = 100 K. Data integration was performed using the SAINT routine^52^ within the Bruker Apex II software package^53^ and a multi-scan absorption correction was performed using SADABS.^54^ The space group was determined using XPREP.^55^ Refinement of the structure was performed using the crystal structure refinement program SHELXL.^56^ All atoms were refined using anisotropic displacement parameters. Reflections corresponding to interplanar distances of *d* < 0.82 Å were excluded from the refinement due to their low intensities.

### Magnetometry

The DC magnetization and AC magnetic susceptibility measurements were performed on single crystals grown by method **1** using a Quantum Design MPMS XL-7 T SQUID magnetometer. The crystals were cut to size (∼2 × 2 × 1 mm) using a razor blade and covered with paraffin wax to protect from degradation by exposure to air and moisture. Field-dependent isothermal magnetization curves, *M*(*H*), were acquired by cycling the field from +*H*_max_ to −*H*_max_ and then back from −*H*_max_ to + *H*_max_. Temperature-dependent AC susceptibility measurements were performed under a DC bias field of *H* = 200 Oe with a superimposed AC magnetic field of *H* = 3.8 Oe oscillating at four frequencies, *f*: 1, 10, 100 and 1000 Hz.

### Neutron scattering

Temperature-dependent neutron powder diffraction was performed using the high-resolution powder diffractometer for thermal neutrons (HRPT) instrument at the Paul Scherrer Institute with a wavelength of *λ* = 1.886 Å from *T* = 200 K to 1.5 K. The samples used for the neutron powder diffraction study were ground-up, twice recrystallized single crystals grown *via* method **1**. Temperature and magnetic-field-dependent small angle neutron scattering experiments were performed on single crystal samples on the V4 instrument at the Helmholtz Zentrum Berlin (HZB). The centimeter-sized single crystals prepared by methods **2** and **3** were sealed in custom-built aluminum sample containers, shown in Fig. S5(c) and (d) (ESI†), under helium atmosphere. The wavelength was *λ* = 3.3 Å and was reached by tilting the velocity selector by 5°.

## Conflicts of interest

There are no conflicts to declare.

## Supplementary Material

MA-006-D4MA00635F-s001

MA-006-D4MA00635F-s002

## Data Availability

Data associated with this article, including magnetometry, neutron powder diffraction and small angle neutron scattering data, are available from the DataverseNL repository at https://doi.org/10.34894/WG9EC9. Crystallographic data for FeCl_3_ have been deposited at the CCDC under Deposition Number 2362658† and can be obtained from https://www.ccdc.cam.ac.uk.
