# Supplementary material for: Magnetic properties of a non-centrosymmetric polymorph of FeCl3
Source: Mater Adv. 2025 Jun 2;6(13):4471–82. doi: 10.1039/d4ma00635f (PMC12142733; doi:10.1039/d4ma00635f)
Supplement: MA-006-D4MA00635F-s001 [file MA-006-D4MA00635F-s001.pdf]

Supplementary Information (SI) for Materials Advances.
This journal is © The Royal Society of Chemistry 2025

Supplemental information for:

Magnetic properties of a non-centrosymmetric polymorph of $FeCl_3$

Joshua J.B. Levinsky[a,b], Ankit Labh[c,d,e], Vladimir Pomjakushin[f], Uwe Keiderling[g], Alexander C. Komarek[h], Li Zhao[h], Jacob Baas[a], Catherine Pappas[c] and Graeme R. Blake[a*]

*Corresponding author: g.r.blake@rug.nl

a. Zernike Institute for Advanced Materials, University of Groningen, Nijenborgh 3, 9747AG Groningen, The Netherlands
b. EaStCHEM School of Chemistry and Centre for Science at Extreme Conditions, University of Edinburgh, Joseph Black building, David Brewster Road, EH9 3FJ, Edinburgh, United Kingdom
c. Faculty of Applied Sciences, Delft University of Technology, Mekelweg 15, 2629JB Delft, The Netherlands
d. Department of Condensed Matter Physics, Charles University, Ke Karlovu 5, 121 16 Praha 2, Czechia
e. TUM School of Natural Sciences, Technical University of Munich, James-Franck-Straße 1, 85748 Garching, Germany
f. Laboratory for Neutron Scattering and Imaging, Paul Scherrer Institut, Forschungsstraße 111, 5232 Villigen, Switzerland
g. Soft Matter and Functional Materials, Helmholtz-Zentrum Berlin für Materialien und Energie GmbH, Hahn-Meitner-Platz 1, 14109 Berlin, Germany
h. Max Planck Institute for Chemical Physics of Solids, Nöthnitzer Straße 40, 01187 Dresden, Germany

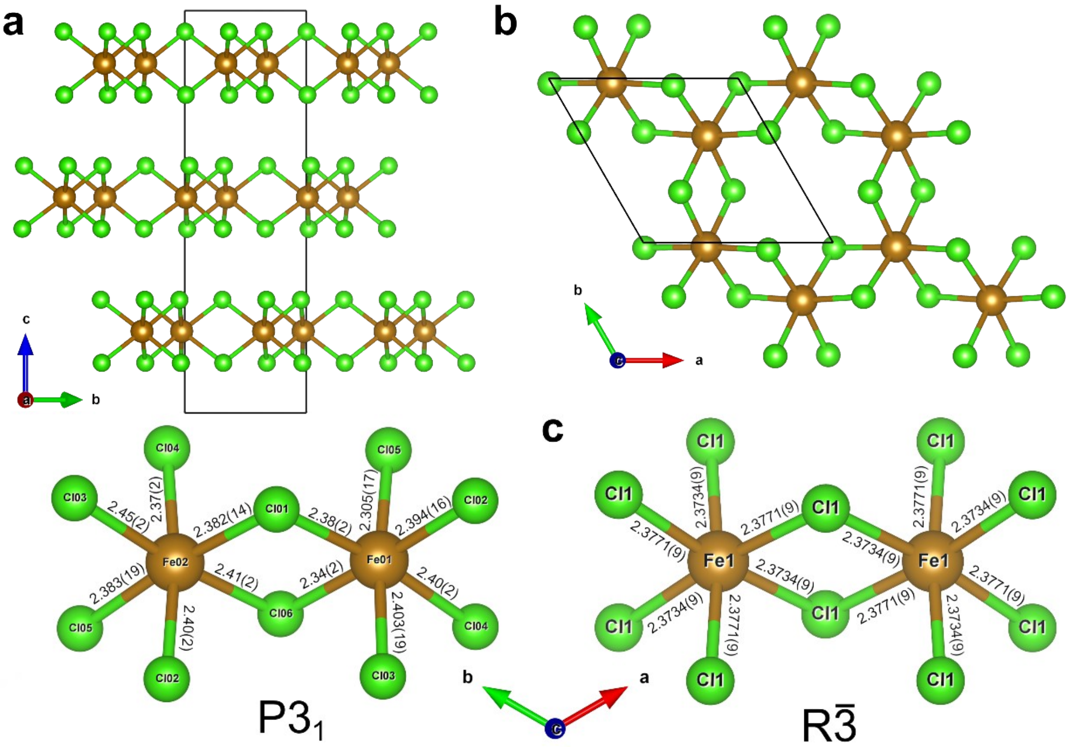

Figure S1 (a) Crystal structure of $FeCl_3$ viewed along the *a*-axis. (b) Single layer of $FeCl_3$ showing the in-plane honeycomb lattice. (c) Fe-Cl bond distances in Å for octahedra belonging to the $P3_1$ (left) and $R\bar{3}_1$ (right) polymorphs of $FeCl_3$ viewed along the *c*-axis.

Table S1 Structure refinement parameters for $FeCl_3$. The numbers in parentheses indicate estimated standard deviations**.**

**Temperature (K)**	100
**Formula**	$FeCl_3$
**Formula weight (g/mol)**	162.2
**Crystal Size (mm^3)**	0.1 x 0.1 x 0.02
**Crystal color**	Black
**Crystal habit**	Hexagonal
**Crystal system**	Trigonal
**Space group**	$P3_1$ (no. 144)
**Z**	6
**Density (calculated) (g/cm^3)**	2.932
**F(000)**	462
**a (Å)**	6.0560(15)
**b (Å)**	6.0560(15)
**c (Å)**	17.354(5)
**α (°)**	90
**β (°)**	90
**γ (°)**	120
**Volume (Å^3)**	551.2(3)
**μ (mm^{-1})**	6.013
**Transmission min/max**	0.0022/0.0230
**θ range (°)**	7.044 to 51.112
**Index ranges**	$-7 \le h \le 7$<br>$-7 \le k \le 7$<br>$-21 \le l \le 20$
**Data/restraints/parameters**	1293/1/68
**GOOF of F^2**	1.155
**# total reflections**	10808
**# unique reflections**	1293
**# observed Fo > 4σ(Fo)**	1013
**R_{int} I > 3σ(I)**	0.0719
**R_{sigma}**	0.0376
**R1 [Fo > 4σ(Fo)]**	0.1426
**R1 [all data]**	0.1605
**wR2 [Fo > 4σ(Fo)]**	0.2930
**wR2 [all data]**	0.2997
**Largest peak/hole (e/Å^3)**	2.31/-1.98
**Flack parameter**	0.4(3)

Table S2 Fractional atomic coordinates and equivalent isotropic displacement parameters (Å^2) for $FeCl_3$. The numbers in parentheses indicate the estimated standard deviations.

Atom	*x*	*y*	*z*	U(eq)
Fe01	0.3550(30)	1.0210(30)	0.5004(4)	0.025(2)
Cl01	0.3310(30)	0.3300(30)	0.5793(7)	0.025(3)
Cl02	0.6770(30)	1.0320(30)	0.5792(7)	0.028(3)
Cl03	0.0350(30)	0.6890(40)	0.5792(8)	0.0314(19)
Fe02	0.0170(20)	0.3450(30)	0.5007(5)	0.040(3)
Cl04	0.3240(40)	0.6780(50)	0.4226(8)	0.044(4)
Cl05	0.6780(30)	1.3140(30)	0.4228(8)	0.0314(19)
Cl06	0.0440(40)	0.0300(40)	0.4223(8)	0.041(4)

Table S3 Anisotropic displacement parameters (Å^2 × 10^3) for $FeCl_3$. The numbers in parentheses indicate the estimated standard deviations.

Atom	U_{11}	U_{22}	U_{33}	U_{23}	U_{13}	U_{12}
Fe01	8(4)	9(4)	47(6)	2(4)	-2(4)	-4(2)
Cl01	35(7)	19(6)	24(5)	1(5)	-8(6)	15(5)
Cl02	13(6)	21(7)	34(7)	5(5)	3(5)	-3(5)
Cl03	23(5)	27(5)	36(4)	1(5)	-3(5)	6(4)
Fe02	43(5)	48(6)	23(4)	2(4)	-1(4)	19(4)
Cl04	41(9)	59(10)	32(7)	7(7)	9(7)	25(8)
Cl05	23(5)	27(5)	36(4)	1(5)	-3(5)	6(4)
Cl06	38(8)	46(8)	36(8)	-1(6)	3(6)	17(7)

Table S4 Bond lengths (Å) for $FeCl_3$. The numbers in parentheses indicate the estimated standard deviations.

Atom	Atom	Length		Atom	Atom	Length
Fe01	Cl01	2.38(2)		Fe02	Cl01	2.382(14)
Fe01	Cl02	2.354(15)		Fe02	Cl02	2.40(2)
Fe01	Cl03	2.403(19)		Fe02	Cl03	2.45(2)
Fe01	Cl04	2.40(2)		Fe02	Cl04	2.37(2)
Fe01	Cl05	2.305(17)		Fe02	Cl05	2.383(19)
Fe01	Cl06	2.34(2)		Fe02	Cl06	2.41(2)

Table S5 Bond angles (degrees) for $FeCl_3$. The numbers in parentheses indicate the estimated standard deviations.

Atom	Atom	Atom	Angle		Atom	Atom	Atom	Angle
Cl01	Fe01	Cl03	89.4(6)		Cl01	Fe02	Cl02	91.8(6)
Cl01	Fe01	Cl04	173.1(7)		Cl01	Fe02	Cl03	90.9(7)
Cl02	Fe01	Cl01	91.0(6)		Cl01	Fe02	Cl05	174.2(9)
Cl02	Fe01	Cl03	90.1(6)		Cl01	Fe02	Cl06	85.5(6)
Cl02	Fe01	Cl04	93.3(8)		Cl02	Fe02	Cl03	90.7(6)
Cl03	Fe01	Cl04	85.1(6)		Cl02	Fe02	Cl06	93.9(6)
Cl05	Fe01	Cl01	95.4(7)		Cl04	Fe02	Cl01	93.3(5)
Cl05	Fe01	Cl02	86.9(6)		Cl04	Fe02	Cl02	173.3(7)
Cl05	Fe01	Cl03	174.3(9)		Cl04	Fe02	Cl03	84.9(7)
Cl05	Fe01	Cl04	90.3(8)		Cl04	Fe02	Cl05	91.2(7)
Cl05	Fe01	Cl06	91.5(7)		Cl04	Fe02	Cl06	90.8(7)
Cl06	Fe01	Cl01	87.1(6)		Cl05	Fe02	Cl02	84.0(6)
Cl06	Fe01	Cl02	177.4(7)		Cl05	Fe02	Cl03	93.1(5)
Cl06	Fe01	Cl03	91.6(5)		Cl05	Fe02	Cl06	90.8(7)
Cl06	Fe01	Cl04	88.8(6)		Cl06	Fe02	Cl03	174.2(7)
Fe01	Cl01	Fe02	93.6(6)		Fe02	Cl04	Fe01	95.9(8)
Fe01	Cl02	Fe02	93.6(6)		Fe01	Cl05	Fe02	95.5(7)
Fe01	Cl03	Fe02	94.1(7)		Fe01	Cl06	Fe02	93.8(7)

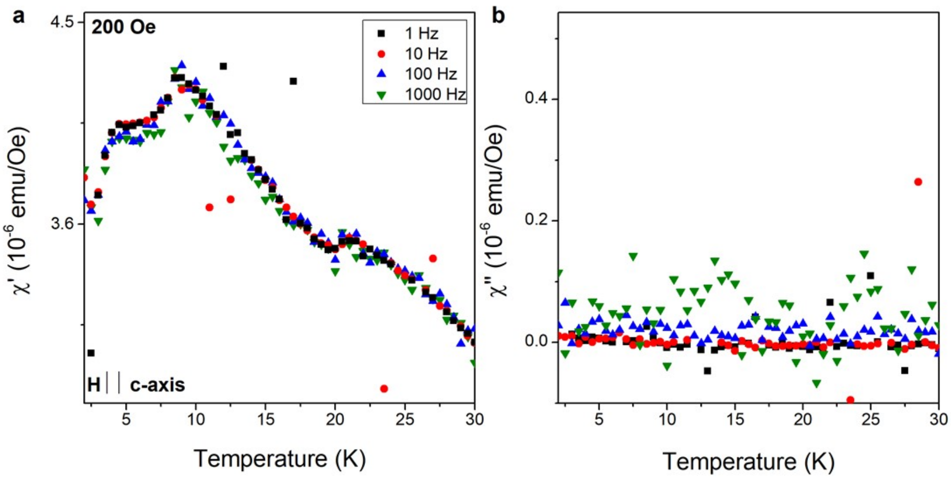

Figure S2 (a) Real and **(b)** imaginary components of the AC magnetic susceptibility plotted against temperature. A DC bias of 200 Oe was applied with a superimposed AC signal of 3.8 Oe oscillating at the four different frequencies indicated in the legend.

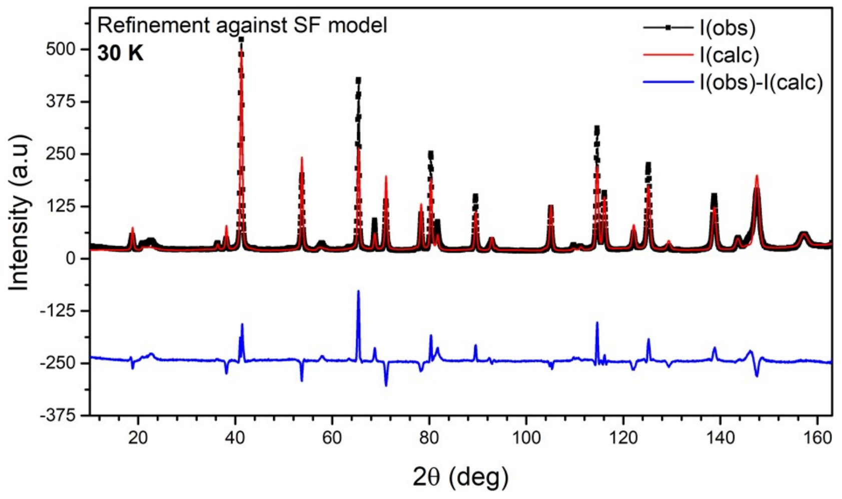

Figure S3 NPD data collected at 30 K fitted using the faulted structure of $FeCl_3$. The difference between $I(calc)$ and $I(obs)$ is plotted in blue.

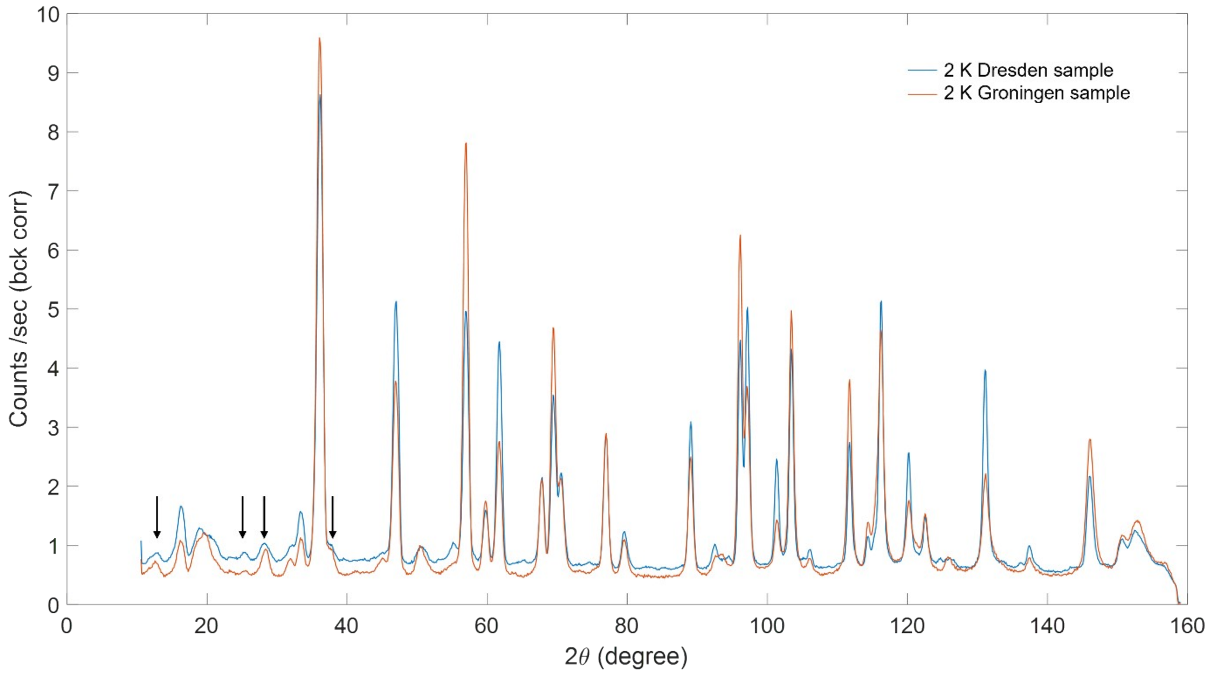

Figure S4 NPD patterns of two $FeCl_3$ samples measured at $T =$ 2 K using the PEARL diffractometer at TU Delft ($\lambda =$ 1.667 Å). The 'Groningen' label refers to the sample grown from 99.6 % pure starting material by the sublimation method that is the focus of the main manuscript, and the 'Dresden' label refers to a second sample grown by chemical vapor transport in a Cl_2 atmosphere (see Experimental Methods for details). The arrows indicate the positions of the magnetic Bragg peaks, demonstrating that the k-vector of (1/2, 0, 1/3) is the same for both samples. Note that the broad feature associated with a high concentration of stacking faults is also observed in both samples at $2\theta \approx$ 20°.

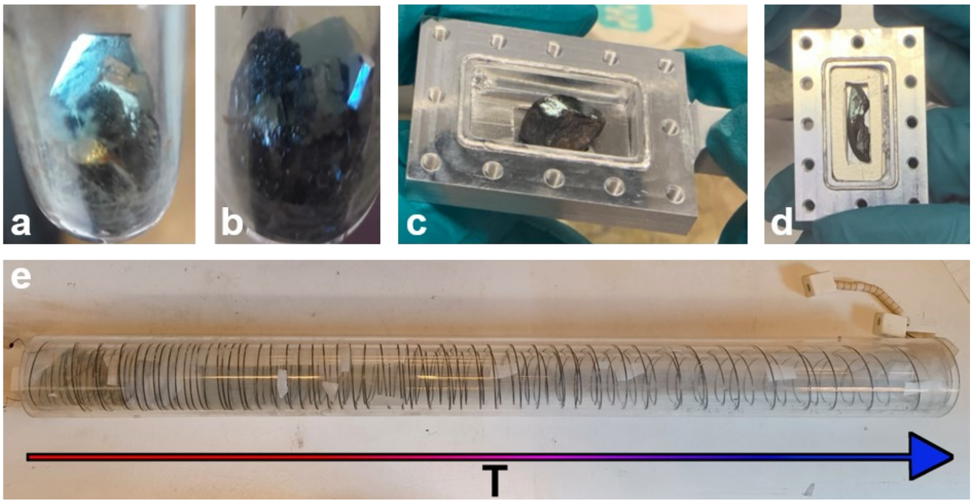

Figure S5 (a)-**(b)** Photographs of a large hexagonally faceted single crystal of $FeCl_3$ grown by method **2**. **(c)**-**(d)** Photographs of open aluminum SANS sample containers containing single crystals grown by methods **2** and **3** respectively. **(e)** Custom-built Bridgman-Stockbarger furnace with an arrow pointing from the high to the low side of the temperature gradient.

References

1 S. I. Troyanov, *Zh. Neorg. Khim.*, 1993, **38**, 1946–1949.
